# Popper’s conjecture with angular slits and twisted light

**DOI:** 10.1038/s41598-023-48915-7

**Published:** 2023-12-07

**Authors:** Neelan Gounden, Jenna Epstein, Pedro Ornelas, Geoff Beck, Isaac Nape, Andrew Forbes

**Affiliations:** School of Physics, University of the Witwatersrand, Private Bag 3, Wits, 2050 South Africa

**Keywords:** Optical physics, Quantum physics

## Abstract

Uncertainty relations are core to both classical and quantum physics, and lend themselves to tests across many degrees of freedom, with structured light emerging as a vibrant tool to harness these degrees of freedom. Here, we test Popper’s conjecture by replacing the traditional spatial and momentum states with angular position and orbital angular momentum (OAM) states of photons, showing that the OAM spectrum for an entangled photon passing through a virtual slit differs from that of a photon passing through a physical slit. To achieve this, we produce two OAM entangled photons, one of which is sent to a slit encoded as a digital hologram, thereby localising its angular position, all the while measuring the OAM of the other. We show that the measured OAM spectrum is limited to that of the initial SPDC photons, independent of the OAM encoded into the slit, consistent with Popper’s viewpoint. Our approach allows us to overcome prior limitations imposed by physical slits and linear momentum, and offers a versatile toolbox for further probes of quantum systems.

## Introduction

Uncertainty relations are core to many branches of physics, spanning space-time bandwidth considerations in ultrafast laser pulses to spatial frequency limits in the analysis of finite apertured optical systems. In keeping with these classical optical examples, it should be noted that Heisenberg’s position-momentum uncertainty principle was itself derived from considerations of an optical microscope^1^ and extrapolated to a more general meaning in the context of quantum mechanics. More recently the emergence of structured light^2^ has seen the renewed testing of these ideas, with orbital angular momentum (OAM)^3–5^ as a key driver. Such so-called twisted light is easily created by imparting an azimuthal ($\phi$) phase twist of $\exp (i\ell \phi )$ to the light, where $\ell$ is an integer, for $\ell \hbar$ of OAM per photon. In recent years OAM has been utilised as an effective tool for demonstrating fundamental tests of quantum mechanics such as higher dimensional Bell inequalities^6^, higher dimensional quantum encryption protocols^7^, and cloning protocols in the optical domain and is therefore a subject of interest. In the quantum realm, OAM entangled states^8,9^ have been used extensively to reveal angle-OAM uncertainty relations^10,11^, while classically entangled^12–14^ forms of OAM structured light have seen the notions of uncertainty, duality, coherence and visibility merge to form new relations^15–19^, even violating uncertainty principles when fair sampling is forsaken^20,21^. The impressive work in using structured light for quantum tests includes Bell-like violations^22^, abstraction of path information and quantum erasures^23^, probing paradoxes^24,25^, distinguishability and symmetry in quantum interference^26^ and many more besides. The reader is referred to the many tutorials^27,28^ and reviews^29–33^ on the topic.

A particularly timeless example is that of Popper’s conjecture^34^, positing that quantum uncertainties are bandwidth limited, a test of fundamental importance^35^ but also of practical relevance to achievable resolution in quantum ghost imaging^36–39^. It has been revisited several times with position-momentum correlated systems but experimental deficiencies, such as lack of control of the source momentum, have maintained a healthy debate on the interpretation of the outcomes^40^. For instance, in previous demonstrations with linear momentum/position, it is challenging to experimentally confirm whether the initial momentum of the photon, prior to passing through the slit, has any influence on the measured uncertainty.

In this work we revisit Popper’s conjecture, deviating from the usual position-momentum basis and use angle-OAM as our degrees of freedom, overcoming some of the previous practical challenges that where imposed by the spatial and momentum degrees of freedom. To achieve this we create two entangled photons by spontaneous parametric downconversion (SPDC) and express the entanglement in the OAM basis, forming a natural higher dimensional Schmidt basis. To test the conjecture, we pass one photon through an angular slit encoded as a computer generated hologram (digital hologram) on a spatial light modulator, measuring the OAM of both at the outcome. The digital version of the slit allows us to precisely control its properties, including imbuing it with OAM to unravel the impact of the source itself having momentum. We outline all the salient experimental considerations and demonstrate the test both in the quantum realm with entangled photons as well as with classically back-projected light, the former testing of the notion of a “virtual” slit and the latter a “physical” slit. The entanglement test shows that indeed the spread in OAM due to the decreased uncertainty in angular position is bandwidth limited to that of the SPDC source, while the classical back-projection reveals the role of the measurement apparatus itself on the outcomes. Our results show that there is momentum spreading due to a virtual slit but bandwidth limited by the entanglement itself, additionally, if the source possesses some initial non-zero OAM (lower-order OAM values, as discussed later on), this has little to no effect on the spread apart from a shift of the OAM spectrum. Our approach allows us to prise apart the interpretation and formalism of quantum mechanics, illustrating that the former is not an adequate explanation of the latter.

## Theoretical considerations

**Figure 1:**
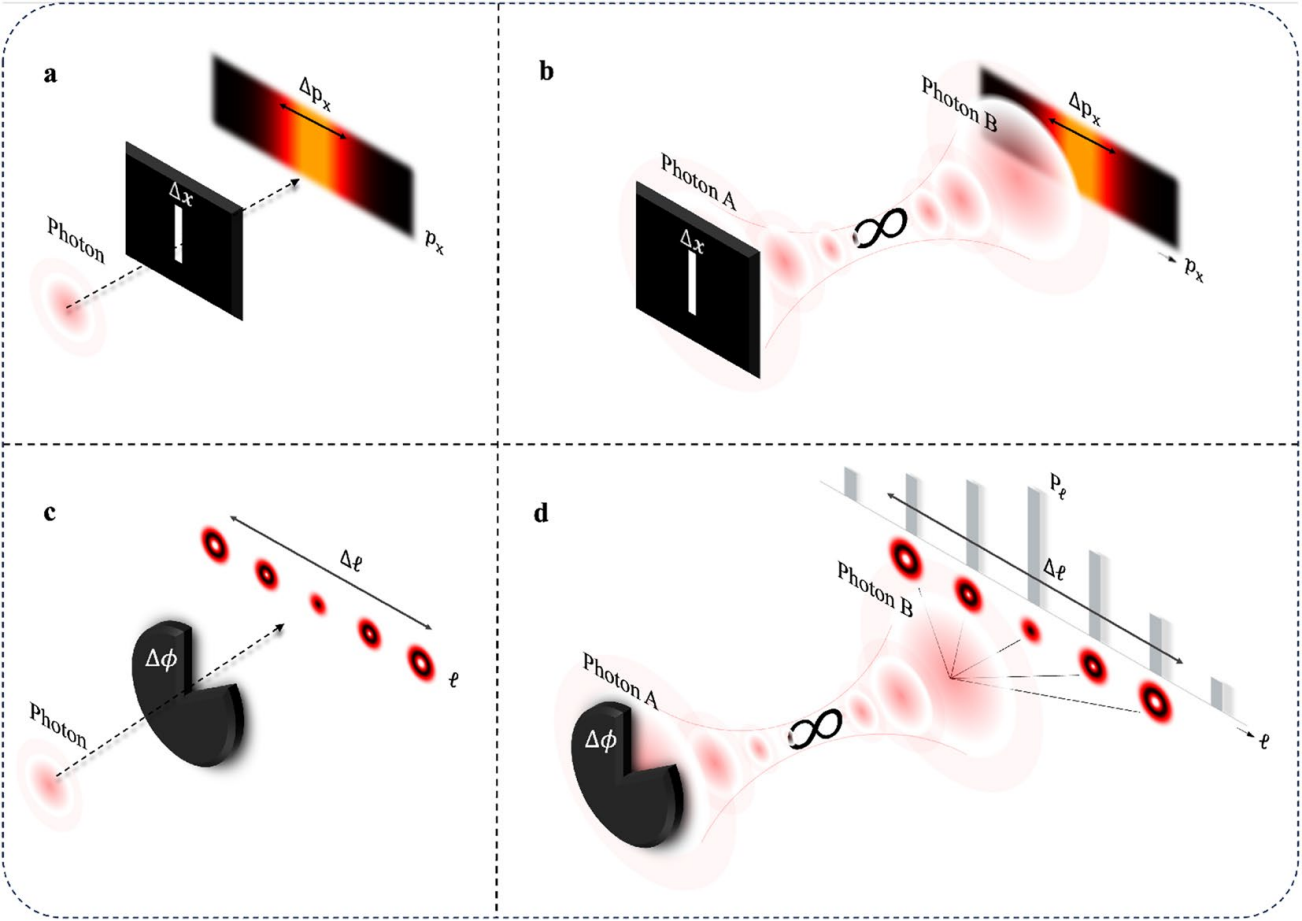
(**a**) The linear case (spatial/momentum) for which the momentum spread is measured. The dashed black line represents a single photon which is incident on a physical linear slit and a linear diffraction pattern is observed, from which a spread in momentum is measured. (**b**) The red rings represents two entangled photons (photon A and B) being generated from a source and propagating (to the left and right respectively), whereby one photon is incident on the linear slit while the momentum of the other photon is measured. (**c**) The angular case (angular position/OAM) for which the OAM spread is measured. The dashed black line represents a single photon which is incident on a physical angular slit and an angular diffraction pattern is observed from which a spread in momentum is measured by decomposing the pattern into orbital angular momentum (OAM) states. (**d**) The angular position/OAM case with entangled photons, for which the spread in OAM is measured. Whereby one photon is incident on the angular slit while the OAM of the other photon is measured.

We begin by introducing the reader to Popper’s conjecture conceptually using the spatial/momentum basis before moving on to the OAM-angle basis of structured photons. As shall be shown, the adaptation is performed by a change in the symmetry of the slit.

### Revisiting Popper’s conjecture

To begin, let us consider a single photon that is traversing a physical slit of width $\Delta x$. Interference patterns will be observed at a distance far away from the slit due to the spread in the momentum of the photon as depicted in Fig. 1a. By narrowing the slit width until it approaches an infinitesimally small value, the width of the momentum spectrum, $\Delta p$, subsequently obtains an unbounded value by virtue of the uncertainty principle

1 $$\begin{aligned} \Delta x\Delta p \ge \frac{\hbar }{2}. \end{aligned}$$

In Fig. 1b we show a similar experiment but now with two entangled photons, where one interacts with the physical slit (Photon A) while the other’s (Photon B) linear momentum is measured. Initially, the momenta of the two photons are conserved, i.e., one photon has momentum *p* while the entangled twin has momentum $-\,p$, resulting in the quantum entangled state,

2 $$\begin{aligned} {|{\Psi _{AB}}\rangle } = \int \Psi _{AB}({\textbf {p}}) {|{{\textbf {p}}}\rangle }_A{|{-{\textbf {p}}}\rangle }_B dp, \end{aligned}$$

where $\Psi _{AB}({\textbf {p}})$ is the normalised two photon wavefunction. Position entanglement follows from the conjugate (Fourier) relation between momentum and position wave-functions, i.e., ${\tilde{\Psi }}_{AB}({{\textbf {x}}}) = {\mathcal {F}} \left( \Psi _{AB}({\textbf {p}}) \right)$. Accordingly, if photon A interacts with a narrowing slit then it experiences an ever larger momentum spread, with the spread in momentum of photon A inferred from that of photon B thanks to the non-local momentum correlations. If the width of the slit in photon A was reduced to an infinitesimally narrow width, $\Delta x \rightarrow 0$, what does it mean for the momentum spread that can be inferred from photon B? Further, is this in any way affected by the momentum of the source, or equivalently, the motion of an observer? Although posed already several decades ago^41^, these questions are debated to this day^34,35,38,42–44^. Popper asserts that there is no influence in the entanglement case, that is, no change in the momentum of Photon B due to is non-local interaction with a “virtual slit” and that any momentum change cannot exceed that of the entanglement source itself.

To answer these questions in a modern context and with full control over the experiment, we recast the question in the language of OAM-angle with digitally controlled angular slits.

### Analogous interpretation using OAM-angle

Analogous to *linear* momentum and *linear* position uncertainty is *orbital angular* momentum ($\ell$) and *angular* position ($\phi$), for what we call OAM-angle uncertainty. Why would we perform a change in our variables? The exchange of linear momentum with OAM provides us with the option of imparting additional OAM onto the photon passing through the angular slit, as if the source did not have zero momentum itself. We depict the OAM equivalent to the traditional picture in Fig. 1c and d for the physical and virtual slit, respectively. In both cases, the linear slit in Fig. 1a and b now become angular slits (pie-like slices) in Fig. 1c and d. The equivalent uncertainty relation is now

3 $$\begin{aligned} \Delta \ell \Delta \phi \ge \hbar /2, \end{aligned}$$

Note that this is the upper limit of the lower bound of the product of $\Delta \ell \Delta \phi$. For details on how this limit can change we refer the reader to Ref. ^45^. To predict the outcome of the experiment depicted conceptually in Fig. 1d, we begin with an initial entangled state expressed in the OAM basis,

4 $$\begin{aligned} {|{\psi }\rangle }_{AB}=\sum _{\ell } c_{\ell } {|{\ell }\rangle }_A {|{-\ell }\rangle }_B. \end{aligned}$$

where $|c_{\ell }|^2$ is the probability for an outcome (two-photon coincidence) of the associated state and can be derived from the entanglement process itself. ${|{\ell }\rangle }_A$ and ${|{\ell }\rangle }_B$ represents an OAM eigenstate for photon A and B respectively. In the case of spontaneous parametric downconversion (SPDC), the workhorse of many quantum optics laboratories, the coefficients are symmetric^46^ about $\ell =0$ and so can be modelled as a normal distribution given by

5 $$\begin{aligned} |c_{\ell }|^2 \propto \exp \left[ -\left( \frac{{\ell }}{\Delta {L}}\right) ^2 \right] , \end{aligned}$$

where $\Delta L$ represents the width of the spectrum in OAM for the initial quantum state (${|{\psi }\rangle }_{AB}$). $\Delta L$ is used as a fitting parameter to fit $|c_{\ell }|^2$ to the measured initial OAM spectrum without any slits present. Let us allow photon A to pass through the physical angular slit, altering the uncertainty in the angular position of this photon. We define our angular slit as

6 $$\begin{aligned} {|{\theta }\rangle }=\int _{0}^{2\pi }\theta (\phi ,\theta _0,\ell _0){|{\phi }\rangle }d\phi \end{aligned}$$

where

7 $$\begin{aligned} \theta (\phi ,\theta _0,\ell _0)= \frac{1}{\sqrt{\theta _0}} {\left\{ \begin{array}{ll} e^{i\ell _0\phi } &{} 0 \le \phi \le \theta _0 \\ 0 &{} \textrm{otherwise} \end{array}\right. } \end{aligned}$$

with the angular coordinate ranging from $0-2\pi$ (centered about $\pi$). Both the size of the slit and OAM encoded into the slit (OAM of an observer relative to the source) can be set through the parameters $\theta _0$ and $\ell _0$, respectively. By encoding OAM into the slit we have the means to impart momentum into the bi-photon system, as if the source did not satisfy $0 = p_A + (-p)_B$ and equivalently $0 = \ell _A + (-\ell )_B$, a point of contention in prior debates, e.g., does the relative motion of an observe or the measurement apparatus change the outcome of the experiment?

To test Popper’s conjecture of the virtual slit we must make an angular position measurement on one photon and an OAM measurement on the other. The joint probability amplitude, resulting from measuring photon A with an angular position of $\theta$ while simultaneously measuring photon B to have an OAM of $\ell '$ can be found from

$$\begin{aligned} \left( {\langle {\theta }|}_A{\langle {\ell '}|}_B \right) {|{\psi }\rangle }_{AB}= \left( {\langle {\theta }|}_A{\langle {\ell '}|}_B \right) \left( \Sigma _{\ell } c_{\ell } {|{\ell }\rangle }_A {|{-\ell }\rangle }_B \right) . \end{aligned}$$

The probability of interest is then

8 $$\begin{aligned} P(\ell )= & {} |({\langle {\theta }|}_A{\langle {\ell '}|}_B){|{\psi }\rangle }_{AB}|^2 \nonumber \\= & {} |c_{\ell } \langle \theta \vert \ell \rangle _A|^2 \nonumber \\= & {} |c_{\ell }|^2\frac{2\theta _0}{\pi }\textrm{sinc}^2{ \left( \frac{\Delta \ell \theta _0}{2} \right) } \nonumber \\\propto & {} \frac{2\theta _0}{\pi } \underbrace{\exp \left( -\left( \frac{{\ell }}{\Delta {L}}\right) ^2 \right) }_\text {entanglement} \,\, \underbrace{\textrm{sinc}^2 \left( \frac{(\ell - \ell _0) \theta _0}{2} \right) }_\text {diffraction} \nonumber \\ \end{aligned}$$

where $P (\ell )$ is the probability of a “click”, a measured joint outcome on photon A and B for an OAM value of $\ell$ on photon B. To finally relate this back to the uncertainty principle we need to define the widths $\Delta \phi$ and $\Delta \ell$. We do so using the usual definition of the uncertainty of a variable (a) as a standard deviation

$$\begin{aligned} \Delta {A}=\sqrt{{\langle {\psi }|}({\hat{A}}-\langle \hat{A\rangle })^2{|{\psi }\rangle }}, \end{aligned}$$

from which we find $\Delta \ell$

9 $$\begin{aligned} \Delta \ell =\sqrt{\Sigma _{\ell }(\ell ^2\cdot P(\ell ))-(\Sigma _{\ell }\ell \cdot P(\ell ))^2}. \end{aligned}$$

Accordingly, $\Delta \phi$ can also be defined as

10 $$\begin{aligned} \Delta \phi =\frac{\theta _0}{2\sqrt{3}}. \end{aligned}$$

**Figure 2:**
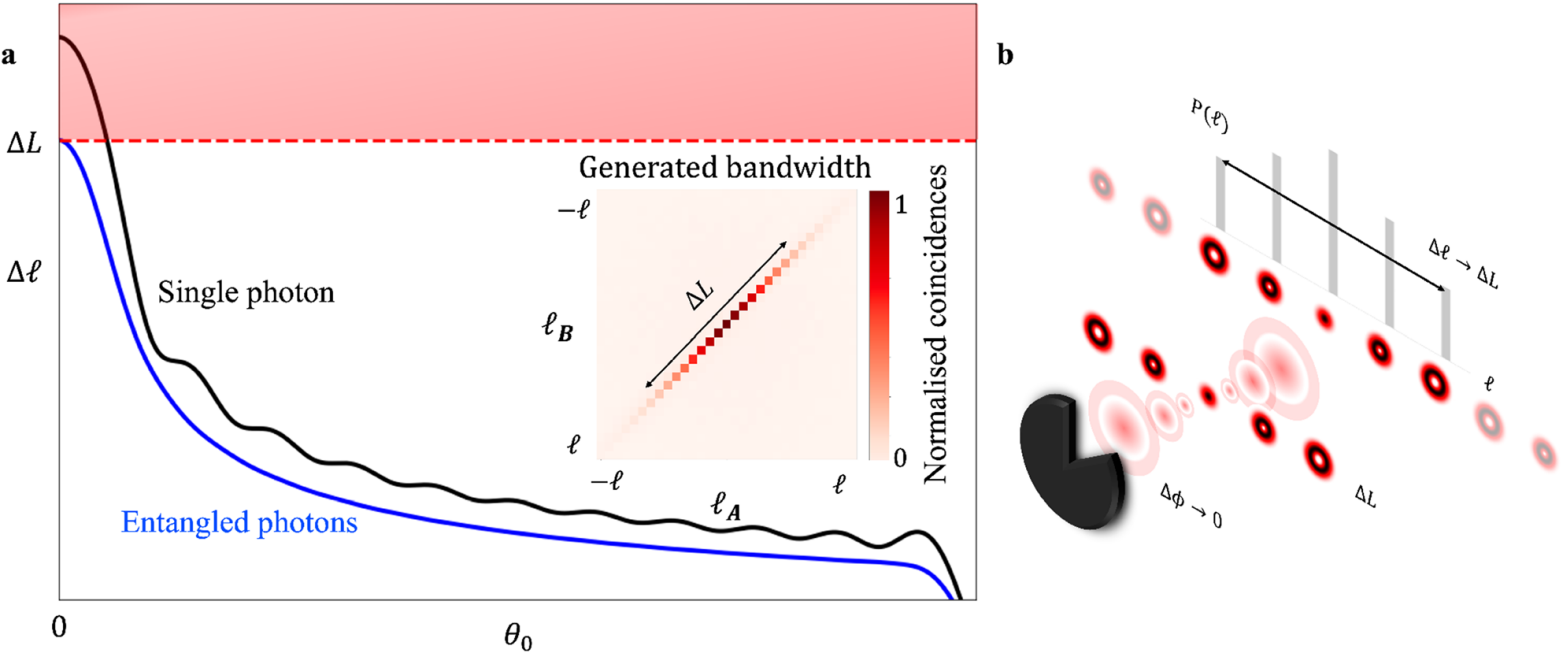
(**a**) The expected results for the uncertainty in OAM ($\Delta \ell$) for a single photon which is passing through an angular slit of width ($\theta _0$) is represented by the black curve (taking into consideration the measurement bandwidth set by the setup). While the blue curve represents the case where an entangled photon passes through a angular slit however the uncertainty in OAM of the entangled twin is measured. The generated bandwidth which represents the initial quantum state, for a given OAM for photon A ($\ell _A$) the probability of measuring photon B with OAM ($\ell _B$) is obtained. The diagonal entries of this matrix are used to obtain the uncertainty in OAM for the initial quantum state $\Delta L$. The uncertainty in OAM for the entangled photon passing through the ‘virtual slit’ will not be able to exceed the uncertainty in OAM for the initial quantum state (represented by the red dashed line). (**b**) If a source produces two entangled photons with an initial,finite spread and one of the entangled photons was incident on a slit of an infinitesimally small width while the OAM spread of the entangled twin is obtained, and is limited to the a finite spread ($\Delta \ell = \Delta L$).

We can unravel the implications of these equations as follows: the probability has two factors, an enveloping Gaussian function courtesy of the entanglement process itself and an oscillating sinc function as a result of the angular diffraction process, with the former bounding the latter. Indeed, the initial OAM entanglement spectrum of $\Delta \ell$ limits the spreading after the slit: without the entanglement factor the spectrum would indeed be unbounded, yet bounded with it. Setting this factor to 1 therefore returns the single photon through a physical slit scenario. We plot both cases in Fig. 2, showing the unavailable bounded region (red) for entangled photons in contrast to the spectrum of the single photon (black) which is not constrained by the same conditions as the entangled photons. In the case of the entangled photons, the width of the OAM spectrum is limited to the width of the initial OAM spectrum. On the other-hand when considering the spectrum produced by a single photon the limited measurement bandwidth leads to the convergence of the width of the spectrum, in order to obtain the spectrum for the single photon $P(\ell )\propto \frac{2\theta _0}{\pi }\textrm{sinc}^2{ \left( \frac{\Delta \ell \theta _0}{2} \right) }$ in conjunction with Eq. (9), resulting in the following for a single photon

11 $$\begin{aligned} \Delta \ell = N\frac{D-\csc \left( \frac{\theta _0 }{2}\right) \sin \left( \frac{\theta _0 D}{2}\right) }{\pi \theta _0 } \end{aligned}$$

where *D* is the total number of OAM modes measured in setup and *N* is a normalisation constant.

According to the Copenhagen interpretation, if the position of a single photon was localised to an infinitesimally small region and the photon’s OAM is measured, it would be predicted that the spread in the OAM of the photon would approach some unbounded value, this would be the case if the measurement system was not taken into consideration and the OAM spectrum was obtained over the entire OAM domain $\ell \in (-\infty ,\infty )$. When the measurement capabilities of the setup are taken into consideration the maximum spread that can be measured is effected since the OAM state for the single photon extends to higher OAM numbers than are measured in the experimental setup. As $\theta _0 \xrightarrow {} 0$, $\Delta \ell$ for the single photon case will approach this $\Delta \ell _{max}$ value which depends on the number of modes being measured in the experimental setup.

The slit width, $\theta _0$, and angular momentum, $\ell _0$, alter the width and position of the resulting OAM spectrum. The shifted position due to $\ell _0$ is a manifestation of the momentum of the source: by applying a non-zero OAM ($\ell _0 \ne 0$) onto the slit the OAM of the photon A ($\ell$) passing through the slit will emerge with a shifted OAM ($\ell + \ell _0$), so that the OAM of photon B will have a spectrum shifted towards $-\ell _0$.

**Figure 3:**
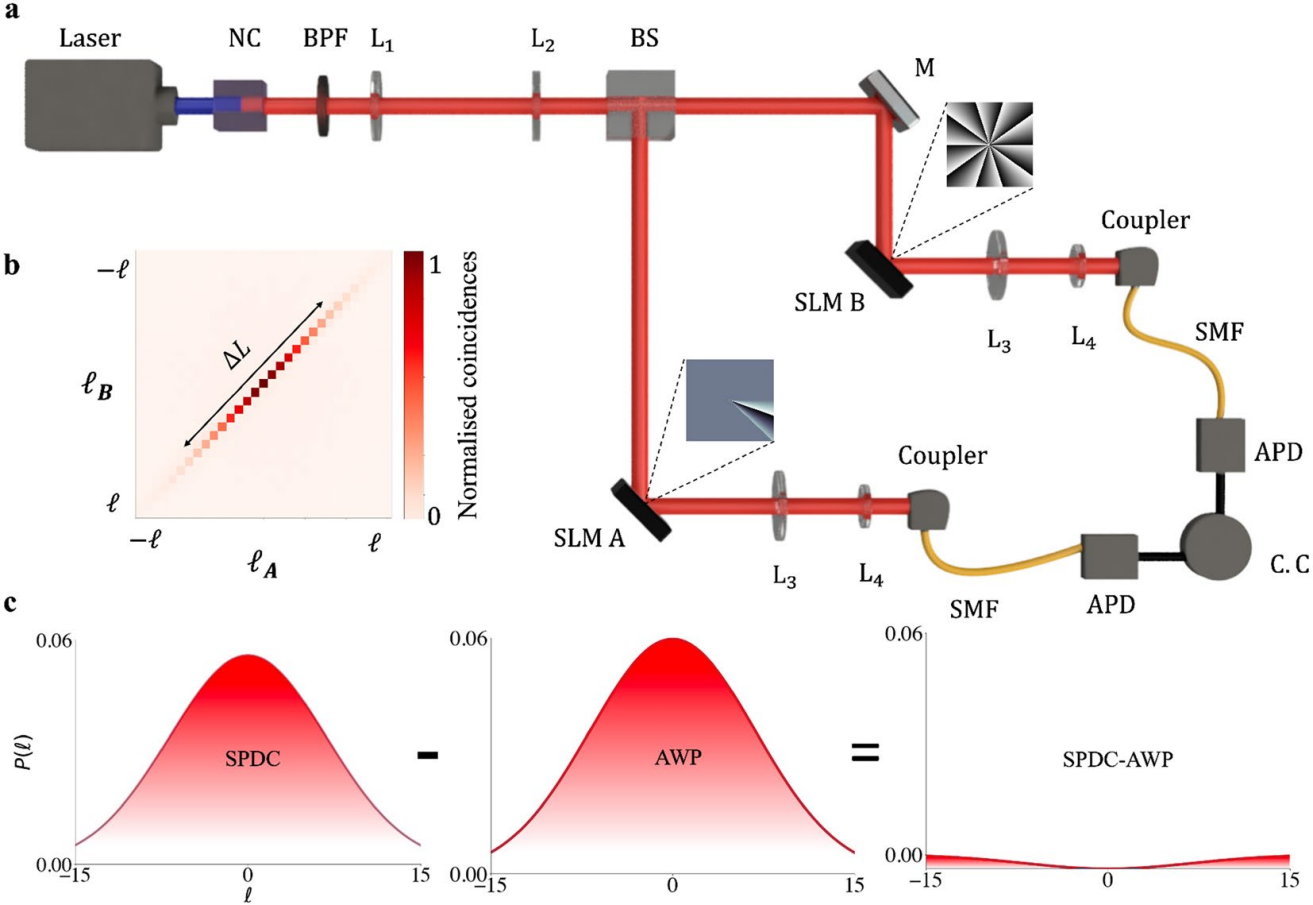
(**a**) A laser (wavelength $\lambda = 355$ nm) produces a pump photon which is incident on a non-linear crystal (NC) which generates two down-converted photons which are entangled, the beam-splitter causes the separation of the entangled photons and the photons follow independent paths, one photon (photon A) is incident on Spatial Light Modulator (SLM) A which has a hologram of an angular slit encoded on it and the other entangled photon (photon B) is incident on SLM B which performs the OAM measurement on photon B, The photons are then incident on their respective couplers which are each connected to Avalanche Photo Diodes (APD) and then finally connected to a coincidence counter (C.C). The band-pass filter (BPF) is used to filter out any pump photons from reaching the beam-splitter. The lens ($L_1-L_4$) are used to image planes in the setup. The mirror (M) is used to ensure that the OAM of photons is not effected by reflections. (**b**) The OAM spectrum of the quantum entangled state produced at the crystal (SPDC spectrum). (**c**) The spectrum labelled ’SPDC’ represents the fit for the initial quantum state from the setup, the spectrum labelled ’AWP’ represents the fit for the measurement capabilities of the setup and is obtained through back-projection. Lastly the spectrum labelled ‘SPDC-AWP’ represents the non-negative difference between the ‘SPDC’ spectrum and the ‘AWP’ spectrum.

## Method

In order to test Popper’s conjecture experimentally, we first generate entangled photons using the setup depicted in Fig. 3a. An ultraviolet $\lambda = 355$ nm photon in a Gaussian $\ell = 0$ state was incident on a Barium Borate (BBO) non-linear crystal (type 1) to produce two $\lambda = 710$ nm photons by spontaneous parametric downconversion (SPDC). The two photons, signal (photon A) and idler (photon B) were separated by a beamsplitter (BS) and imaged with two lenses to the plane of the spatial light modulators (SLMs), one in each path. One SLM was encoded with a digital version of the angular slit (SLM A) while the other (SLM B) performed projective measurements in the OAM basis, with example holograms for each shown as insets. The resulting outcomes were collected in single mode fibre (SMF) with by a demagnifying telescope, detected by single photon Avalanche-Photo Diodes (APDs), and measured in coincidence with a gating time of 2 ns and an integration time of 60 s. In order to compensate for isotropic noise, which leads to coincidence detections which have not been obtained from the entangled photons in the system, an isotropic state was used to model our experimental state^47^

12 $$\begin{aligned} \rho =p{|{\psi }\rangle }{\langle {\psi }|}+\left( \frac{1-p}{d^2} \right) {\mathbb {I}}_{d^2}, \end{aligned}$$

where *p* represents the probability of measuring a pure state. $\rho$ is the density matrix of dimension $d \times d$ with a pure portion, ${|{\psi }\rangle }$, and a mixed portion, ${\mathbb {I}}_{d^2}$. A pure state corresponds to purity $p=1$ while a maximally mixed state corresponds to purity $p=0$, while the dimensionality can be obtained by calculating the schmidt mode number, $d \equiv K = (\Sigma _l |c_l|^2)^{-1}$, or by direct measurement^48^. An example of the OAM spectrum without any angular slits is shown in Fig. 3b. From this initial spectrum we can deduce the parameter $\Delta L= 6.6 \pm 0.3$. Fitting the model of Eq. (12) returns $d = K_\text {SPDC} \approx 24$ with a purity of $p = 0.185$ which was used for each experimental OAM spectrum produced in order to compensate for the isotropic noise, this allows for the experimental OAM spectra to collectively be fit to the theoretical model. Note that the maximum purity achievable in an experiment falls off sharply with increasing dimensionality, which for 24 dimensions is $p_\text {max} = 0.271$. In other words, the noise in the experiment was low and with an average quantum contrast of $\text {Q} = 9.912$^48^.

Much of the prior debate was centred on the nature of the measurement system and so before beginning with the main tests, we demonstrate the role of the measurement system in the outcome of the process. To do this we make use of Klyshko’s advanced wave picture (AWP)^49^, which invokes the equivalence of momentum conservation at the SPDC source to that of backward travelling waves under reflection at the crystal. Experimentally this is easily executed by replacing one of the photon detectors (say photon B) with a source of classical light, allowing the light to follow the path backwards from the detector of Photon B, reflecting off the crystal face as a mirror, and then travelling forward along the path of photon A to its detector. Such a procedure has demonstrated the utility of the AWP in many different quantum systems, where quantum results can be predicted using classical light^27,49–51^. We employ it to obtain the OAM bandwidth of the measurement system itself, with the outcome shown visually in Fig. 3c). We see that the number of detectable OAM modes is largely dictated by the measurement system itself, with the classical OAM width of $\Delta L_\text {AWP}=6.1\pm 0.3$ comparable to that of SPDC, $\Delta L_\text {SPDC}=6.6\pm 0.3$, with only a small deviation between the two. In other words, most of what is measured is the measurement system itself! This is not particular to our set-up but a general property of such SPDC experiments with OAM. Interestingly, a calculation of the dimensionality in the two systems as measured by the Schmidt numbers^46^ suggests that $K_\text {SPDC} \le K_\text {AWP}$, i.e., the SPDC spectrum will fall within the measurable limits of the setup.

## Results

**Figure 4:**
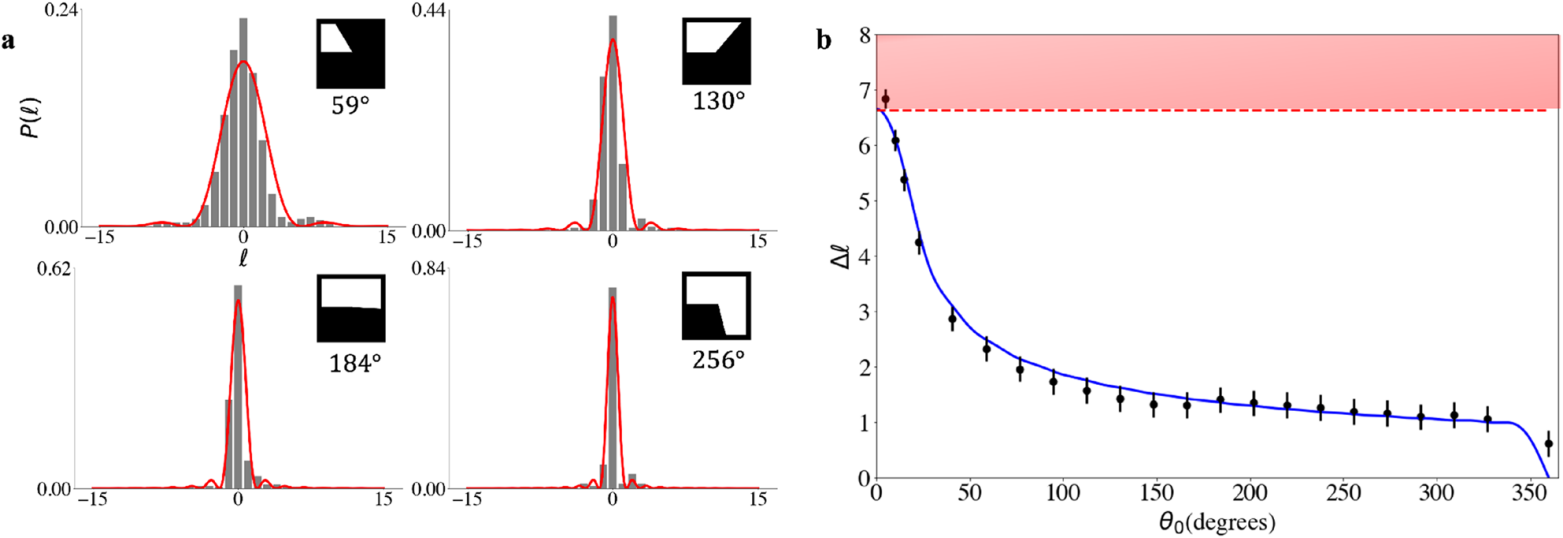
(**a**) Normalised OAM spectra are generated from coincidence counts (gray bar plot) while being compared to the theoretical model (red curve). (**b**) Using the normalised OAM spectra (for photon B) which are being generated the widths of these spectra are calculated and plotted against the width of the angular slit which photon A passes through (black scatter plot points), while being compared to the widths of the theoretical spectra (blue curve). The red dashed line represents the width of the initial quantum entangled state (SPDC spectrum) shown in Fig. 3b.

To test the limits on $\Delta \ell$ given some choice of $\theta _0$, we vary the angular slit on SLM A in the full range $\theta _0 \in [0,360^{\circ }]$ in 22 equally spaced intervals while maintaining $\ell _0 = 0$ for no initial source momentum, with the results summarised in Fig. 4. In Fig. 4a we show the measured OAM spectrum (grey bars) together with the theoretically expected spectrum shape (red curve) from Eq. (8) for example angular slits corresponding to $\theta _0 = 59^{\circ }$, $130^{\circ }$, $184^{\circ }$ and $256^{\circ }$, all in excellent agreement. The resulting experimental widths of the spectra were calculated to be $\Delta \ell = 2.3 \pm 0.2$, $1.4 \pm 0.2$, $1.4 \pm 0.2$ and $1.2 \pm 0.2$, respectively.

**Figure 5:**
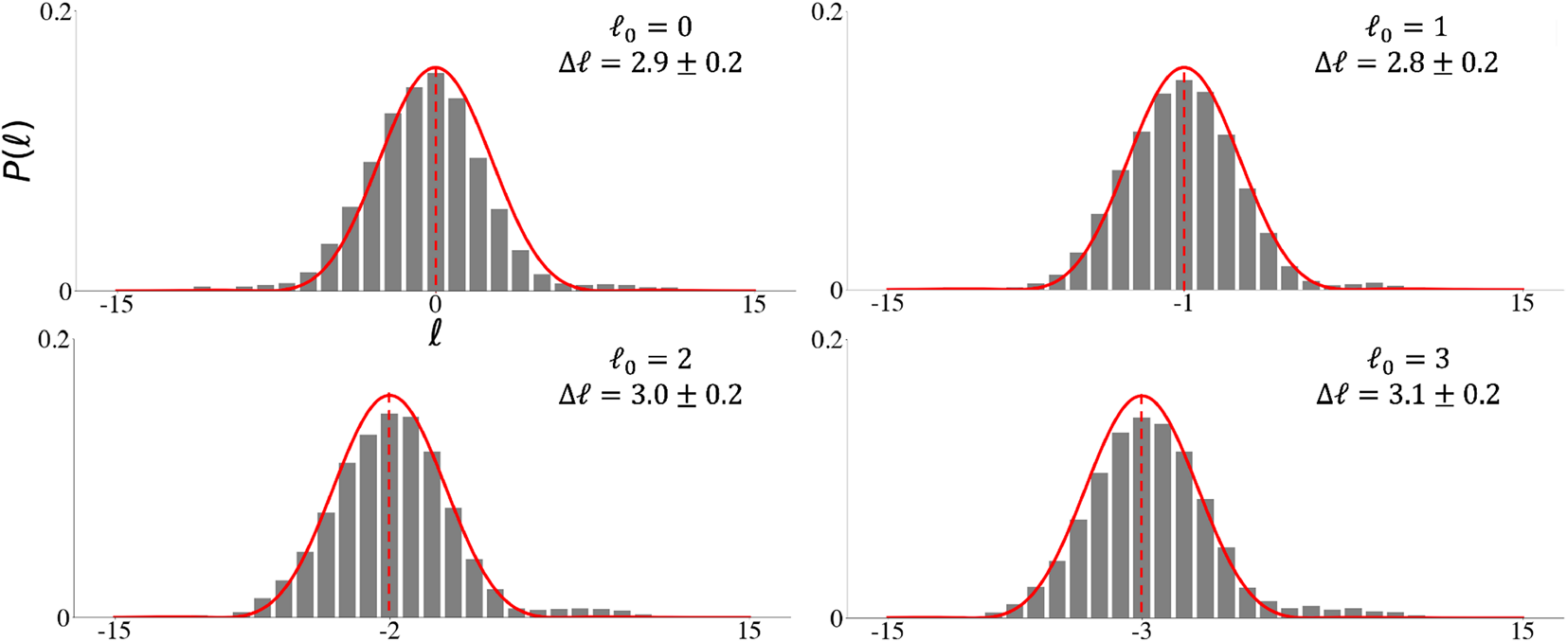
Applying a non-zero OAM mode within the slit of $45^{\circ }$ which photon A passes through, causing additional OAM to be imparted onto photon B leading to the corresponding shifts in the OAM spectra measured for photon B. The width of the spectra measured are equal in value to within experimental error (only for lower-order OAM modes encoded within the slit).

The result of all such measurements is shown in Fig. 4b as data points together with the theoretical prediction using Eq. (9), both in excellent agreement. From the results we see that as the width of the angular slit decreases the width of the OAM spectrum increases, roughly inversely proportional to one another. But when the width of the angular slit approaches an infinitesimally small value, $\theta _0 \xrightarrow {} 0$, so the uncertainty in OAM converges to a finite value, $\Delta \ell \xrightarrow {} \Delta L = 6.6 \pm 0.3$, i.e., bandwidth limited by the initial quantum entangled state (SPDC state). Decreasing the angular slit size to smaller values than $5^{\circ }$ lead to a smaller amount of photons being sampled for the OAM measurement, which leads to the domination of noise in the OAM spectrum. Allowing for this source of error, we see that the width of the observed OAM spectra cannot exceed that produced at the crystal, indicated by the red dashed line in Fig. 4b. Our results reveal that the correlation between the entangled photons limits the spread of the OAM, preventing $\Delta \phi \Delta \ell$ from approaching $\hbar /2$ at small angles, and thus that the OAM spread generated through SPDC cannot be exceeded by performing a OAM measurement of a photon passing through a “virtual slit”. In Fig. 5 we show the impact of a non-zero OAM mode ($\ell _0$) being encoded onto the slit (changing the OAM of the observer relative to the source). We see that indeed the centre of the OAM spectrum shifts towards $-\ell _0$, but that the width of the spectrum is unaltered and with a maximum relative error between the widths of 0.097 can be determined between the spectra centered at $\ell _0=1$ and $\ell _0=3$ with $\Delta \ell =2.8$ and $\Delta \ell =3.1$ respectively. The relative momentum of the observer (changing the source OAM) does not alter the band-limited property (for lower-order OAM values), however a shift in the width of the OAM spectrum is expected if the OAM of the observer is large relative to the source.

## Discussion

Popper conjectured that no additional momentum spread can be added to that of the initial momentum spread produced at the source. From our results we have shown that this conjecture must indeed be true. While for a single photon passing through a slit, the OAM spread tends towards some unbounded value, only limited by the measurement apparatus, we find that the momentum spread of the photon passing through the ’virtual slit’ is limited by the initial momentum spread of the quantum entangled state produced at the source. Furthermore, our results also indicate that the momentum spread of the photon passing through the ’virtual slit’, is independent of whether or not the source possessed some initial non-zero momentum.

In the context of Popper’s thought experiment, a central confusion has been a conflation of the formalism of quantum mechanics and the Copenhagen interpretation itself. According to Popper^41^, the Copenhagen interpretation of quantum mechanics posits that the momentum spread of photon B will approach an unbounded value because of the momentum correlations to photon A; a similiar observation would occur if a single photon passed through the physical linear slit and the momentum of this photon was measured. A counter argument is that the Copenhagen prediction is whatever the formalism predicts (perhaps an example of what Home describes as “epistemology without ontology”). While this may seem sensible, it is not necessarily the case when discussing the interpretation of a mathematical formalism, rather than the formalism itself. After all, the core of the formalism is shared between all interpretations. The role of the formalism is to provide a shared mathematical backbone, while interpretation explains what these mathematics relate to in the real world (sometimes supplementing a common formalism with additional mathematical structure).

In order to follow Popper’s claims we should turn to the source he cites as the most robust exposition of the Copenhagen viewpoint: David Bohm’s 1951 book^52^. In this work, Bohm states that making a position measurement with some uncertainty $\Delta x$ must result in a momentum uncertainty $\gtrsim \frac{\hbar }{\Delta x}$. He then reinforces this by noting a single quantum being confined by a slit, and subsequently deflected, is a manifestation of the Heisenberg uncertainty principle. In this he is not alone, many authors of popular textbooks present the same idea to this day^53–61^. Though, only some of these authors explicitly relate the uncertainty principle to ‘knowledge’ of a system’s properties, it is still common to describe uncertainty as “limiting our knowledge” for incompatible observables in individual measurements. Furthermore, Bohm asserts that complementarity *requires* that, if the position of a particle becomes more definite, then the momentum must become less definite. Thus, we can see that the Copenhagen position holds that photon A has its momentum scattered in a measurement of *x* with precision $\Delta x$. This, in turn, constitutes a measurement of photon B because of, as Bohm puts it, the “indivisible unity of the world”. More concretely, Copenhagen asserts that if the two entangled photons share a quantum state, they share an indivisible existence, matching the positivism which under-girds Copenhagen via Bohr and Heisenberg^41^. Thus, we can see that the Copenhagen viewpoint, as elucidated by Bohm, requires that photon B must feel some effect of measuring A, we are after all, measuring their combined state. Our measurement does not completely “collapse” the state (as Copenhagen would have it) but it never the less incurs a reduction from the original wave function, making the photon positions “more definite”. This seems to be sufficient to argue that the Copenhagen view has that our measurement of A constitutes a position measurement of B. Combined with the above view of the uncertainty relations and complementarity, we must now admit that the momentum of B should be scattered in turn as its value must have become “less definite”. One could argue that this scatter happens, but should be limited by the initial beam-width. However, this would depend upon a different understanding of the uncertainty relations and complementarity than the one described above. All of this seems enough to confirm that Copenhagen predicts momentum broadening, whether or not this is directly related to the uncertainty principle. The outcome of the experiment is then in Popper’s favour. This means one cannot maintain an interpretation of entanglement whereby the participant objects are actively connected, responding non-locally to local operations on the sub-systems. The positivist/Copenhagen interpretation of the unfactorisable state is therefore untenable in light of this experiment.

For future improvements, the use of hard/square apertures leads to unnormalisable OAM distributions, to combat the situation, soft/Gaussian apertures can be used to truncate the OAM spectra (this position state for the photon produced a soft slit which leads to $\psi _{\ell }$ also being truncated thus eliminating the normalisation problem^10^, increase the bandwidth of modes produced at the source so that the spread of modes will occur over a large range of OAM values and the limitation of the spread of the OAM spectra in the results will be more pronounced at smaller angles, this can be done by magnifying the beam size on the non-linear crystal. Increasing the signal to noise ratio especially for measurements taken for small and large aperture sizes would lead to an improvement in the results, this can be done by increasing the integration time over which the measurements are taken, decreasing the gating time and lowering the power of the pump beam on the crystal.

We have shown that structured light is an effective tool for testing underlying principles in quantum mechanics, overcoming previous challenges by judiciously selecting the degrees of freedom of photons. As opposed to using non-linear crystals to generate OAM for the entangled photons, the use of meta lenses could be used to structure the quantum light^62^ and could possibly be used in future research. Crucially, our work demonstrates the impact of measurements (observer) and source characteristics of quantum states in high dimensional Hilbert spaces, requiring consideration of the source bandwidth, entanglement of the quantum particles and the measurement system along with its bandwidth capabilities. The complexity of the characterisation the quantum state increases alongside an increase in dimensionality of the system. These parameters have an impact on emerging quantum- imaging and communication protocols, such as improving the resolution of quantum imaging and encoding information in higher dimensional quantum states. Therefore addressing foundational issues such as Popper’s conjecture is crucial for further developments.

## Conclusion

We have revisited Popper’s conjecture in the context of OAM-angle uncertainty with entangled photons, and should inspire the further use of structured photons in quantum tests. We see that the OAM of the photon that does not pass through the slit is indeed broadened by the reduced angular uncertainty of the photon that does pass through the slit, but that this broadening is limited to the initial OAM spectrum of the two-photon state. We outlined the benefits of our experimental implementation, namely, digital control over both the angular position and OAM of the source, thereby resolving prior points of contention. We include a classical back-projected experiment that reveals the nature of the measurement process itself, a point that we are sure will solicit discussion and debate. Finally, we provide a full theoretical treatment and discussion of its meaning, thereby covering both the mathematical framework and its interpretation. In summary, we implement the experiment with a new set of variables while making a more careful examination of what the experiment means.

## Data Availability

The datasets used and/or analysed during the current study available from the corresponding author on reasonable request.
